# From Chickens to Humans: The Importance of Peptide Repertoires for MHC Class I Alleles

**DOI:** 10.3389/fimmu.2020.601089

**Published:** 2020-12-14

**Authors:** Jim Kaufman

**Affiliations:** ^1^ School of Biological Sciences, Institute for Immunology and Infection Research, University of Edinburgh, Edinburgh, United Kingdom; ^2^ Department of Pathology, University of Cambridge, Cambridge, United Kingdom

**Keywords:** epistasis, Avian immunology, immunopeptidomics, B locus, BF-BL region, minimal essential major histocompatibility complex

## Abstract

In humans, killer immunoglobulin-like receptors (KIRs), expressed on natural killer (NK) and thymus-derived (T) cells, and their ligands, primarily the classical class I molecules of the major histocompatibility complex (MHC) expressed on nearly all cells, are both polymorphic. The variation of this receptor-ligand interaction, based on which alleles have been inherited, is known to play crucial roles in resistance to infectious disease, autoimmunity, and reproduction in humans. However, not all the variation in response is inherited, since KIR binding can be affected by a portion of the peptide bound to the class I molecules, with the particular peptide presented affecting the NK response. The extent to which the large multigene family of chicken immunoglobulin-like receptors (ChIRs) is involved in functions similar to KIRs is suspected but not proven. However, much is understood about the two MHC-I molecules encoded in the chicken MHC. The BF2 molecule is expressed at a high level and is thought to be the predominant ligand of cytotoxic T lymphocytes (CTLs), while the BF1 molecule is expressed at a much lower level if at all and is thought to be primarily a ligand for NK cells. Recently, a hierarchy of BF2 alleles with a suite of correlated properties has been defined, from those expressed at a high level on the cell surface but with a narrow range of bound peptides to those expressed at a lower level on the cell surface but with a very wide repertoire of bound peptides. Interestingly, there is a similar hierarchy for human class I alleles, although the hierarchy is not as wide. It is a question whether KIRs and ChIRs recognize class I molecules with bound peptide in a similar way, and whether fastidious to promiscuous hierarchy of class I molecules affect both T and NK cell function. Such effects might be different from those predicted by the similarities of peptide-binding based on peptide motifs, as enshrined in the idea of supertypes. Since the size of peptide repertoire can be very different for alleles with similar peptide motifs from the same supertype, the relative importance of these two properties may be testable.

## Introduction

Molecules encoded by the major histocompatibility complex (MHC) of jawed vertebrates play central roles in immune responses as well as other important biological processes (1). Among these molecules are the classical class I molecules, which are defined by presentation of peptides on the cell surface, high and wide expression and high polymorphism. There are also non-classical class I molecules that lack one or more of these properties; in this report, only the classical class I molecules will be considered and will be abbreviated MHC-I.

MHC-I molecules bound to appropriate peptides on a cell surface are ligands for thymus-derived (T) lymphocytes through the T cell receptor (TCR) composed of α and β chains (along with the co-receptor CD8), with the outcome generally being death of the target cell through apoptosis (2). The cytotoxic T lymphocytes (CTLs) are important agents for response to infectious pathogens (particularly viruses) and cancers. The repertoire of TCRs is formed by somatic mutational mechanisms in individual cells and is vast and cross-reactive, so that in principle any MHC molecule bound to any peptide could be recognized (3). In fact, selection of T cells in the thymus strongly affects the TCR repertoire, but, to a first approximation, it is the polymorphism of the MHC molecules along with self-peptides that determines thymic selection, presentation of peptides, and thus immune responses (4).

However, many MHC-I molecules are also ligands for natural killer (NK) cells through a variety of NK receptors (NKRs), with the potential outcomes including cytokine release and target cytotoxicity (2). Analogous to T cell education based on the MHC molecules and self-peptides present in an individual, the responses of NK cells depend on the particular MHC molecules present during development, a phenomenon referred to as education, licencing, or tuning (5). Both NKRs and MHC-I ligands are polymorphic, with the interactions of particular receptors with particular ligands varying markedly in strength. Since the MHC and the regions encoding NKRs are located on different chromosomes, the genetic result is epistasis, which in humans and mice affects infectious disease, autoimmunity, and reproduction. Indeed, there appears to be antagonistic selection between immune responses and reproduction in humans (6).

MHC-I molecules (7) generally bind short peptides, 9–11 amino acids in length, along a groove between two α-helices above a β-pleated sheet. The peptides are tightly bound at the N- and C-termini by eight highly-conserved amino acids in pockets A and F, so that longer peptides bulge in the middle. Specificity of binding to different MHC-I alleles arises from peptide interactions with the polymorphic amino acids that line the groove, often with deeper pockets B and F being most important, but with other pockets being important in some alleles. The important pockets typically bind just one amino acid or a few amino acids with side chains that have very similar chemical properties, although some promiscuous pockets allow many different amino acids. The particular amino acids generally allowed to bind in the important pockets, the so-called anchor residues, give rise to peptide motifs for MHC-I alleles. Many alleles have been grouped into several supertypes (8) based on similarities in peptide motifs and in polymorphic amino acids lining the pockets. Some motifs are quite stringent in their requirements while others are more permissive, leading the concepts of fastidious and promiscuous MHC-I alleles with differently sized peptide repertoires (9).

TCRs recognize the side chains of peptide residues that point up and away from the peptide-binding groove, mostly in the middle of the peptide (10). It has long been known that the particular peptides bound to MHC-I molecules could influence interaction with inhibitory NKRs (11–14), which eventually was refined to NKR interaction with side chains near the end of the peptide (typically residues 7 and 8 of a 9mer) (15, 16). Moreover, both viral and bacterial peptides have been reported to affect recognition by activating NKRs (17, 18).

Among the questions that will be considered in this report are the extent to which the size of the peptide repertoire may influence the binding NKRs, and the extent to which MHC-I alleles within a supertype have the same sized peptide repertoire. In order to approach these questions, it is appropriate to review what is known about peptide repertoires, beginning with chicken class I molecules.

## The Chicken MHC: A Simple System for Discovery

**Figure 1:**
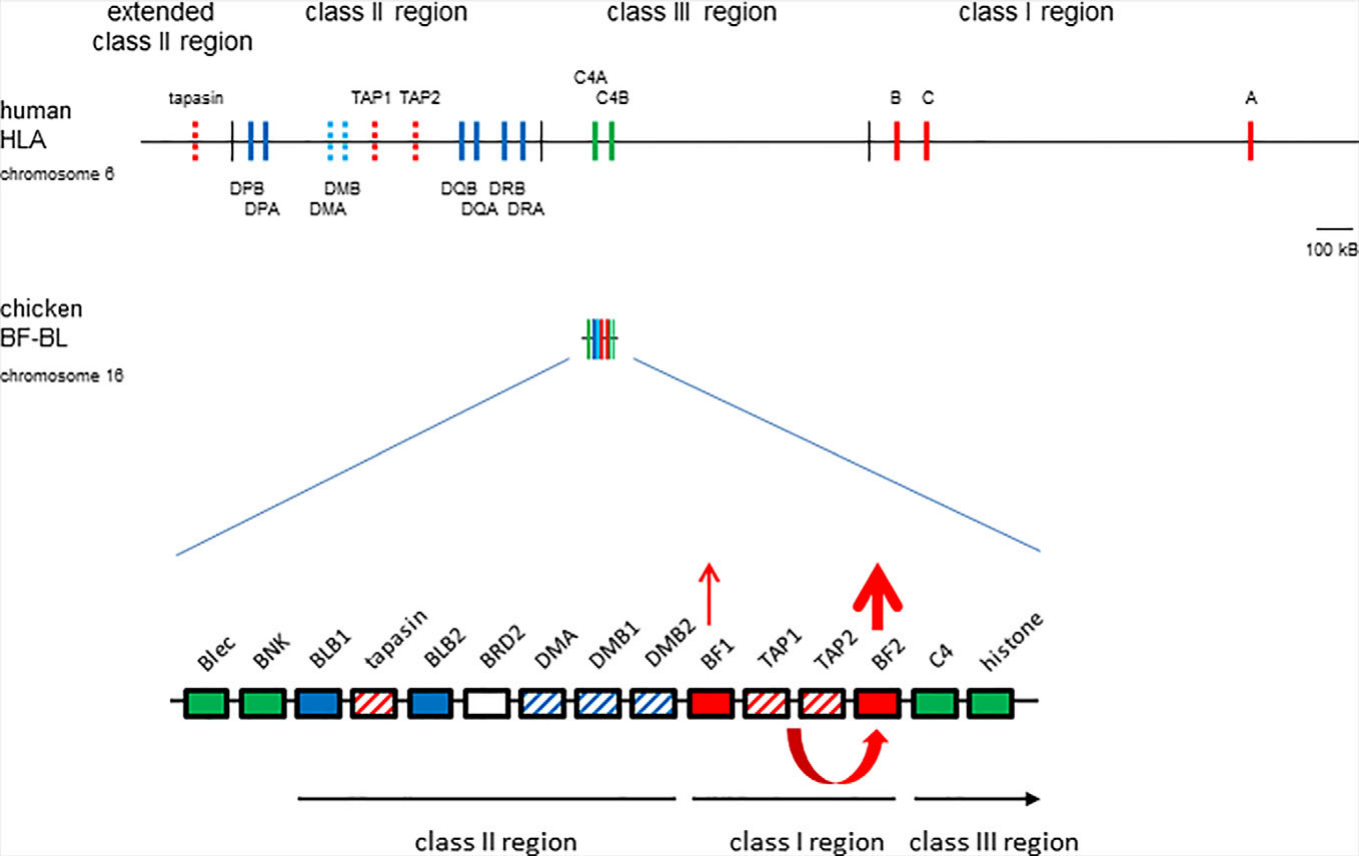
The chicken MHC (BF-BL region) is smaller and simpler than the human MHC (HLA locus), with a single dominantly-expressed MHC-I molecule due to co-evolution with peptide-loading genes. Colored vertical lines or boxes indicate genes, with names above; thin vertical lines indicate region boundaries, with names above or below; location is roughly to scale, with the length of approximately 100 kB indicated. Thickness of arrows pointing up indicate level of expression, co-evolution between the TAP genes and the BF2 class I gene indicated by a curved arrow beneath the genes. Genes from the class I system, red; the class II system, blue; the class III or other regions, green; solid colors indicate classical genes while striped colors indicate genes involved in peptide loading. Figure from (9).

The vast majority of what is known about the MHC and MHC molecules was discovered in humans and biomedical models like mice (1). In typical placental mammals (**Figure 1**), the MHC is several megabase pairs (Mbp) of DNA with hundreds of genes, separated into haplotype blocks by several centimorgans (cM) of recombination. The few MHC-I genes located in the class I region are separated from the few class II genes in the class II region by the class III region which contains many unrelated genes. Some genes involved in the class I antigen processing and presentation pathway (APP) are also located in the MHC, including two genes for inducible proteasome components (LMPs or PSMBs), two genes for the transporter for antigen presentation (TAP1 and TAP2) and the dedicated chaperone and peptide editor tapasin (TAPBP). However, these class I APP genes are located in the class II region and are more-or-less functionally monomorphic (19–21), working well for nearly all loci and alleles of MHC-I molecules. In humans, the three loci of MHC-I molecules may not be interchangeable: HLA-A and -B present peptides to CTLs with only some alleles acting as NKR ligands, while HLA-C is less well-expressed and mostly functions as an NKR ligand (22, 23). There is also evidence that HLA-A and -B may do different jobs, since HLA-B is more strongly associated with responses to rapidly evolving small (RNA) viruses, while HLA-A may be more involved with large double-stranded DNA viruses (24).

In contrast, the chicken MHC is small and simple (**Figure 1**), and evolves mostly as stable haplotypes (9, 25). The BF-BL region of the B locus is less than 100 kB and contains two MHC-I genes (BF1 and BF2) flanking the TAP1 and TAP2 genes, with the TAPBP gene sandwiched between two class II B genes nearby, and with the class III region on the outside. There is evidence only for historic recombination within this region, with no examples of recombinants from over 20,000 informative progeny in deliberate mating, although there is clear recombination just outside (in the so-called TRIM and BG regions) (26–28). As a result, alleles of these strongly-linked genes stay together for long periods of time, so that the APP genes are all highly polymorphic and co-evolve with the BF2 gene (9, 29). As an example, the peptide translocation specificity of the TAP is appropriate for the peptide binding specificity of the BF2 molecule encoded by that haplotype (30, 31). Apparently as a result, the BF2 molecule is far more expressed and also more polymorphic than the BF1 molecule (32, 33). Thus far, the evidence is that the BF2 molecule presents peptides to CTLs, while BF1 functions as a ligand for NK cells (34).

This simplicity of the chicken MHC can make it easier to discover phenomena that are difficult to discern in the more complicated MHC of humans and other placental mammals. For example, there are many examples of strong genetic associations of the B locus (and in some cases, the BF-BL region) with responses to economically-important diseases, including Marek’s disease caused by an oncogenic herpesvirus, infectious bronchitis caused by a coronavirus and avian influenza (9, 35). In contrast, the strongest associations of the human MHC are with autoimmune diseases, with the strongest associations with infectious disease being with small viruses like HIV (1). One hypothesis for this perceived difference is the fact that the human MHC has a multigene family of class I molecules which confer more-or-less resistance to most viral pathogens (reading out as weak genetic associations), while the chicken MHC has a single dominantly-expressed class I molecule, which either finds a protective peptide or not (reading out as strong genetic associations) (9, 36).

Other examples of discovery from the apparent simplicity of the chicken MHC will be described below, but it has become clear that other aspects of the avian immunity may be very complex, for instance the chicken NKR system.

## Promiscuous and Fastidious Class I Alleles in Chickens

One of the discoveries that was facilitated by the presence of a single dominantly-expressed chicken class I molecule is an apparent inverse correlation between peptide repertoire and cell surface expression, along with strong correlations with resistance to infectious diseases. Some so-called promiscuous BF2 alleles bind a wide variety of peptides but have a relatively low expression on the cell surface cell, while other so-called fastidious BF2 alleles bind a much more limited variety of peptides but have higher cell surface expression (9, 32, 37, 38).

It is not clear whether there is a hierarchy or two general groups of alleles, or to what extent the cell surface expression levels are exactly an inverse of the peptide repertoire. The analysis of expression level by flow cytometry is quantitative, but the exact levels vary for different cell types. The peptide repertoires are far more difficult to quantify, with even immunopeptidomics that fairly accurately counts numbers of different peptides by mass spectrometry suffering from the drawback that the abundance of any given peptide is laborious to establish definitively. However, for certain well-studied standard B haplotypes, the peptide-motifs based on gas phase sequencing and on immunopeptidomics, as well as the pockets defined by crystal structures, give qualitative rationales for the peptide repertoires (9, 32, 37–41). The peptide translocation specificities of the TAP alleles from the few haplotypes examined provide additional support (30, 31).

The high expressing fastidious alleles typically bind peptides through three positions with only one or a few amino acids allowed (32, 39–41). For instance, the BF2 allele from the B4 haplotype (BF2*004:01) binds almost entirely octamer peptides with three acidic residues: Asp or Glu at positions P2 and P5, and Glu (with very low levels of hydrophobic amino acids) at position P8, which fits the basic amino acids forming the so-called pockets B, C, and F in wire models and the crystal structure. BF2*012:01 binds octamer peptides with Val or Ile at position P5 and Val at position P8, but with a variety of amino acids at position P2, which is an anchor residue as seen by structure. BF2*015:01 binds peptides with Arg or Lys in position P1, Arg in position P2 and Tyr (with very low levels of Phe and Trp) at positions P8 or P9. In fact, these BF2 alleles with fastidious motifs can bind a wider variety of peptides *in vitro* than are actually found on the cell surface (31, 39); the TAP translocation specificities are more restrictive than the BF2 peptide binding specificities.

In contrast, it would appear that a variety of binding mechanisms can lead to low expressing alleles with promiscuous motifs. BF2*021:01 has certain positions with small amino acids leading to a wide bowl in the centre of the binding groove, within which Asp24 and Arg9 can move, remodelling the binding site to accommodate a wide variety of 10mer and 11mer peptides with co-variation of P2 and Pc-2 (two from the end), along with hydrophobic amino acids at the final position. Interactions between P2, Pc-2, Asp24, and Arg9 allow a wide range of amino acid side chains in the peptide, with at least three major modes of binding (37, 38). Analysis of peptide translocation in B21 cells shows the specificity is less stringent than the BF2*021:01 molecule (31). In another mechanism, BF2*002:01 binds peptides with two hydrophobic pockets for P2 and Pc, but the pockets are wide and shallow, allowing a variety of small to medium-sized amino acid side chains (38). BF2*014:01 also has two pockets, accommodating medium to large-sized amino acid side chains at P2 and positive charge(s) at Pc (38). Binding many different hydrophobic amino acids allows a promiscuous motif, since hydrophobic amino acids are so common in proteins.

Another interesting feature of chicken class I molecules is C-terminal overhang of peptides outside of the groove. In placental mammals, one of the eight invariant residues that bind the peptide N- and C-termini is Tyr84, which blocks the egress of the peptide at the C-terminus. However, in chickens (and all other jawed vertebrates outside of placental mammals), the equivalent residue is an Arg (42, 43) and this change allows the peptide to hang out of the groove, as has been found in crystal structures of BF2*012:01 and 014:01 (28, 30). At least one low expressing class I allele with an otherwise fastidious motif shows lots of such overhangs (C. Tregaskes, R. Martin and J. Kaufman, unpublished), suggesting that the TAP translocation specificity (or perhaps the TAPBP peptide editing) controls the extent to which overhangs are permitted. Interestingly, the equivalent position in class II molecules is also Arg, allowing most peptides to hang out of the groove, with some of these overhangs recognized by TCRs (40, 43, 44). Thus, the presence of such overhangs may be a third mechanism for chicken class I promiscuity, and may affect both TCR and NKR recognition, as do peptide sidechains within the groove in humans (10, 16).

The reason for the inverse correlation of peptide repertoire with cell surface expression is not clear. Among the possibilities are biochemical mechanisms, which are highlighted by the fact that all chicken BF2 alleles have nearly identical promoters, and that the amount of protein inside the cell does not differ much, but that the amount that moves to the cell surface is more for fastidious than promiscuous alleles (31). Thus, the amount of time associated with the TAPBP and TAP in the peptide-loading complex (PLC) could be a mechanistic reason. Another potential biochemical mechanism might be stability and degradation; promiscuous alleles from cells are overall less stable than fastidious alleles in solution, but pulse-chase experiments of *ex vivo* lymphoctyes show no obvious difference in turn-over (31). As a second reason, the correlation could arise from the need to balance effective immune responses to pathogens and tumours with the potential for immunopathology and autoimmunity. A third possibility is the need to balance negative selection in the thymus with the production of an effective naïve TCR repertoire: more peptides presented would mean more T cells would be deleted, but since TCR signal depends on the number of peptide-MHC complexes, lower class I expression would mean fewer T cells would be deleted (9, 45). If true, the expression level would be the important property, since it would mirror the need for an effective T cell repertoire.

What makes this inverse correlation so interesting is the association with resistance and susceptibility to economically-important pathogens. A correlation with low cell surface expression was first noticed for resistance to the tumours arising from the oncogenic herpesvirus that causes Marek’s disease, and later understood to correlate with a wide peptide repertoire (9, 36–38). Important caveats include the fact that the association of the B locus with resistance to Marek’s disease, while still true for experimental lines, have not been found for current commercial chickens (46–48); an explanation may be the fact that poultry breeders have strongly enriched for low expressing class I alleles in their flocks so that the MHC no longer has a differential effect (C. Tregaskes, R. Martin and J. Kaufman, unpublished). Another caveat may be that there are various measures of the progress of Marek’s disease, and the BF-BL region correlations may not be the same for all of them. A third caveat is that the BF-BL region is composed of strongly-linked genes, so that the gene (or genes) responsible for resistance are not yet definitively identified; an example is the evidence for the effect of the BG1 gene (49). An important counter to these caveats is that there is evidence that MHC haplotypes with low-expressing class I alleles confer resistance to other infectious viral diseases, including Rous sarcoma, infectious bronchitis and avian influenza (9, 50–52). Importantly, there is little recognized evidence that the high expressing alleles provide important immune benefit to chickens.

## Promiscuous and Fastidious MHC Molecules in Humans: Generalists and Specialists

Having clear evidence of the inverse correlation of cell surface expression level with peptide repertoire of chicken BF2 alleles and infectious disease resistance, it was natural to ask whether these relationships are fundamental properties of class I molecules, as opposed to some special feature of chicken class I molecules. Such evidence for human HLA-A and B alleles with hints towards potential mechanisms was not hard to find.

Progression of human immunodeficiency virus (HIV) infection to frank acquired immunodeficiency disease syndrome (AIDS) is one of the best examples for an association of infectious disease with the human MHC. Some HLA alleles lead to fast progression and death, while others result in very slow progression, for which the individuals can be called elite controllers (53, 54). The number of peptides from the human proteome predicted to bind four HLA-B alleles was compared to odds ratio for AIDS, finding that the most fastidious alleles were the most protective. Although the correlation with disease resistance was the reverse of what was found for chickens (45), flow cytometric analyses of these four alleles on *ex vivo* blood lymphoid and myeloid cells showed that these human class I molecules had the same inverse correlation between peptide repertoire and cell surface expression as in chickens (38).

A mechanism of resistance by such elite controlling HLA-B alleles has been reported: the presentation of particular HIV peptides to CTLs which the virus can mutate to escape the immune response, but only at the cost of much reduced viral fitness. For such alleles, the virus is caught between a rock and a hard place (55, 56). The protection to the human host afforded by binding and presenting such special peptides led to a hypothesis (9, 38), in which the promiscuous class I alleles act as generalists, providing protection against many common and slowly evolving pathogens (as in chickens), while the fastidious alleles act as specialists, with particular alleles providing protection against a given new and quickly evolving pathogen (as in humans). There are some caveats to this story. One is that the predictions are only a reflection of reality, based on benchmarking the predictions made by such algorithms against experimental data from immunopeptidomics (57). Another is that other explanations are possible; a study calculating the number of peptides predicted for class II alleles concluded that promiscuous alleles would appear based on the number of pathogens in particular environments (58).

**Figure 2:**
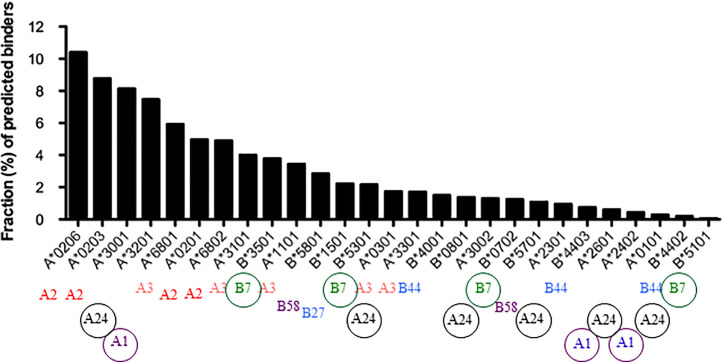
The predictive peptide repertoires for 27 common HLA-A and -B alleles [from (59), Copyright 2013. The American Association of Immunologists, Inc.] compared to the supertypes of these alleles [from (8)] show that the peptide motifs do not correlate well with peptide repertoire for some supertypes.

Another study determined the number of peptides from dengue virus predicted to bind 27 common HLA-A and -B alleles, concluding that there is a wide variation in peptide repertoire that is inversely correlated with stability (59), similar to what was found for chicken class I molecules. Three of the four HLA-B alleles analyzed in the human proteome study were also analyzed in this dengue study and followed the same hierarchy (**Figure 2**). Interestingly, more HLA-B alleles were found at the fastidious end of the spectrum and more HLA-A alleles were found at the promiscuous end, particularly HLA-A2 variants. It would appear that HLA-A and B alleles have a range of peptide repertoires but perhaps not as wide as in chickens. The fastidious chicken class I molecules typically have three fastidious anchor residues compared to two for human class I molecules, while the promiscuous HLA-A2 variants each allow two or three hydrophobic amino acids compared to five or more for BF2*002:01. Unlike chicken MHC-I molecules, peptide overhangs from human MHC-I molecules are relatively rare and require major re-adjustments of peptide-binding site, such as movement of α-helices that line the groove (60, 61), so this is not likely to be a general mechanism for promiscuity in humans.

## Mechanisms for Establishing Peptide Repertoires in Human Class I Molecules

The question arises whether the peptide motif determines the peptide repertoire for human class I molecules, given that the APP genes for human class I molecules are more-or-less functionally monomorphic so that all class I alleles will get a wide and promiscuous set of peptides and peptide editing. As mentioned above, supertypes of MHC-I molecules have been defined based on shared peptide motifs and on amino acids lining the pockets of peptide binding sites (8). A comparison of the peptide repertoires presented in the Dengue study (59) with such supertypes (**Figure 2**) shows that some peptide motifs correlate well with peptide repertoire (for example A2, A3, etc); for example, the alleles falling within the A2 supertype are all found at the promiscuous end of the repertoire. However, alleles from other supertypes (for example A1, A24, and B7), are found across the spectrum of repertoires. Thus peptide motifs do not equate with peptide repertoires, giving the possibility of discriminating between the two designations in terms of contribution towards disease.

A study on the dependence of cell surface expression of HLA-B alleles on TAPBP (also known as tapasin) may give a clue as to the discrepancy between peptide motif and peptide repertoire (62). There are many reports of particular pairs of alleles varying in TAPBP-dependence, and positions in the α2 and α3 domains have been identified that affect this dependence. A hierarchy of dependence was described for 27 HLA-B alleles (63), and a rough correlation with the hierarchy of peptide repertoire was found: fastidious alleles were by-and-large more dependent of TAPBP for cell surface expression, while promiscuous alleles were not (9). Such dependence would fit with the stability of class I molecules mentioned above: Peptide editing by TAPBP leads to the fastidious class I molecules retaining only the peptides that have the highest affinity, while promiscuous class I molecules would bind and move to the cell surface with any peptide with a minimal affinity. Moreover, the authors concluded that tapasin-independent alleles were linked to more rapid progression from HIV infection to death from AIDS (63).

Interestingly, this dependence of TAPBP correlated with the ease of refolding with peptides *in vitro* (in the absence of TAPBP), with both human and chicken promiscuous alleles refolding more easily (38, 62). Whether chicken class I alleles have the same dependence *in vivo* is not yet clear, since TAPBP is highly polymorphic, with the TAPBP and BF2 alleles in each haplotype likely to have co-evolved (64).

## HLA-C and BF1: Flies in the Ointment?

The fact that there are relationships of cell surface expression, peptide repertoire and resistance to infection disease both for BF2 in chickens and for HLA-A and -B in humans suggested that these are fundamental properties of MHC-I molecules. However, the evidence for HLA-C in humans and BF1 in chickens, which have some intriguing similarities, may not fit this emerging paradigm (9).

HLA-C is the result of an ancient gene duplication of HLA-B, but the two differ in several important ways (22, 23). Both HLA-B and -C molecules are polymorphic, are up-regulated upon inflammation, and bind and present peptides to αβ T cells. However, HLA-B molecules are expressed at the RNA, protein and cell surface levels as well as HLA-A molecules. HLA-B molecules are major CTL ligands on virally-infected cells, but some alleles carrying the Bw4 epitope on the α1 helix of the peptide-binding domain are also recognized by NKRs, specifically the killer immunoglobulin receptors with three extracellular domains (3D KIRs).

In contrast, HLA-C molecules are expressed at a low RNA level and are found at about 10% of the level of HLA-A or -B molecules on the surfaces of cells where all three loci are expressed. However, they are also expressed on extravillous trophoblasts (EVT) in the absence of HLA-A and -B molecules. HLA-C alleles are known as important NKR ligands, by carrying either C1 or C2 epitopes on the α1 helix of the peptide-binding domain, which are recognized by different KIRs with two extracellular domains (2D KIRs). Moreover, different HLA-C alleles have different RNA and cell surface protein levels, for which those with higher expression are correlated with slow progression from HIV infection to AIDs, and with some evidence to suggest that this correlation is due to recognition by CTLs (65, 66). There have been no experiments reported to explicitly test the relationship of peptide repertoire and cell surface expression of HLA-C alleles, but the determination of cell surface expression has been reported to be very complex, including effects of promoters, miRNA, assembly, stability and peptide-binding specificity (67).

Much less is known about the chicken BF1 gene, but it has some similarities to the HLA-C. BF1 molecules are expressed at a much lower level than BF2 molecules, at the level of RNA, protein and antigenic peptide (32, 33). There are far fewer alleles of BF1 than BF2, with ten-fold less BF1 RNA found in most haplotypes and with some haplotypes missing a BF1 gene altogether peptide (32, 33). BF1 is also thought to be primarily an NKR ligand (34), and most BF1 alleles carry a C1 motif on the α1 helix of the peptide-binding domain (68, 69). Examination of sequences suggests that most BF1 alleles have similar peptide-binding grooves, with the few examples of other sequences likely to have been due to sequence contributions from the BF2 locus (C. Tregaskes, R. Martin and J. Kaufman, unpublished). An unsolved question is how BF1 alleles interact effectively with the highly polymorphic TAP and TAPBP alleles, for instance accommodating the very different peptides from translocated by TAPs in different haplotypes. Perhaps the typical BF1 molecule is highly promiscuous, but there are few data for either peptide repertoire or cell surface expression among BF1 alleles.

## The Other Side of the Coin: Receptors on Natural Killer Cells

An enormous body of scientific literature describes the very complex evolution, structure and function of NKRs and NK cells in primates and mice (2, 70, 71). Two kinds of NKRs are found in humans, lectin-like receptors found in the natural killer complex (NKC) and the KIRs in the leukocyte receptor complex (LRC). The KIRs are a highly polymorphic multigene family with copy number variation, and share the human LRC with other immunoglobulin-like receptors, including leukocyte immunoglobulin-like receptors (LILRs) and a single receptor for antibodies (Fcμ/αR or CD351). Some of these transmembrane receptors have cytoplasmic tails with immune-tyrosine inhibitory motifs (ITIMs), others have basic residues in the transmembrane region which allow association with signaling chains bearing immune-tyrosine activating motifs (ITAMs), and a few have both. The polymorphic NKRs interact with polymorphic MHC-I molecules, 2D KIRs with HLA-C and 3D KIRs with certain HLA-A and HLA-B alleles. As mentioned above, the interactions of the particular alleles present in LRC and MHC, which are on different chromosomes, lead to differing outcomes, which read out as genetic epistasis with effects on immunity, autoimmunity and reproduction.

In chickens, almost all of the known immunoglobulin-like receptors related to KIRs are found on a single microchromosome, different from the one on which is found the MHC (72, 73). These chicken immunoglobulin-like receptors (ChIRs) include those with activating, inhibitory and both motifs (ChIR-A, -B, and -AB), and 1D, 2D, and 4D extracellular regions. Sequencing studies suggest there can be haplotypes with few ChIR genes in common, suggesting both copy number variation and polymorphism (74–76). However, a gene typing method for 1D domains suggested relatively stable haplotypes, with only some examples of recombination during matings (77). The only molecules that have clear functions are many ChIR-AB molecules that bind IgY, the antibody isotype that acts somewhat like IgG in mammals (78–80). It seems very likely that there are both activating and inhibitory NKRs among these ChIRs, but thus far no data for NKR function. Whether such putative NKRs recognize BF1, BF2, or both is as yet unknown, and whether there is epistasis between the ChIR and MHC microchromosomes is untested.

Among the lectin-like NKR genes located in the NKC in humans and mice are one or more NKR-P1 genes (also known as NK1.1, KRLB1, or CD161) paired with the lectin-like ligands (LLT1 in humans and Clr in mice). In chickens, there are only two lectin-like genes located in the region syntenic to the NKC, and neither of those appears to encode NKRs; one is expressed mainly in thrombocytes (81, 82). However, there is a pair of NKR-P1/ligand genes in the chicken MHC (25, 83), known as BNK (sometimes identified as Blec1) and Blec (sometimes identified as Blec2). The receptor encoded by the highly polymorphic BNK gene was assumed to interact with the nearly monomorphic Blec gene, but a reporter cell line with one BNK allele was found not to respond to BF1, BF2 or Blec, but to spleen cells bearing a trypsin sensitive ligand (84, 85). A trypsin-sensitive ligand on a particular chicken cell line was found to reproduce the result with the reporter cells, but the nature of that ligand remains unknown (E. K. Meziane, B. Viertlboeck, T. Göbel and J. Kaufman, unpublished). Possibilities include other lectin-like genes in the BG region or the Y region of the MHC microchromosome (28, 86).

The effect of peptide repertoire of class I molecules on NK recognition has not carefully examined in either humans or chickens, but some speculations may be worth considering. A wider peptide repertoire may increase the number (although unlikely the proportion) of peptides with appropriate amino acids to affect binding to KIRs and ChIRs, both at the level of response and potentially at the level of education (licensing or tuning), including the recently described phenomenon of cis-tuning (87). However, the increase in breadth of peptide repertoire may be balanced by the decrease of cell surface expression of the class I molecules, which may mean that peptide repertoire may not exert an enormous effect on inhibitory NK responses. In contrast, any increase in peptide repertoire may allow additional pathogen peptides to be recognized by activating NKRs. A special consideration are C-terminal overhangs, which may be particularly frequent in at least some alleles of chicken class I molecules. Such C-terminal overhangs in human class II molecules can directly affect T cell recognition (44), so it is possible that NKR interactions could also be affected.

## Conclusions

The simplicity of the chicken MHC has allowed discoveries of phenomena that were harder to discern from analysis of the more complicated MHC of humans and mice (such as the existence of promiscuous and fastidious MHC-I alleles), but comparison between the immune systems of chickens and mammals has been fruitful (as in the development of the generalist-specialist hypothesis). For human MHC-I molecules, peptide motifs (as identified by supertypes) can be separated from peptide repertoire (as defined thus far by peptide prediction), but their impact on NKR recognition has not been tested. Moreover, careful analysis of Pc-1 and Pc-2 residues in promiscuous versus fastidious alleles with respect to peptide repertoire has not yet been carried for either humans or chickens. Given that the most basic understanding of NKR recognition in chickens has yet to gained, the importance of C-terminal peptide overhang from chicken MHC-I alleles for NKR recognition or NK function has not yet been assessed. Thus, it is clear that there is much work to do to understand NK cell function in chickens, and how that function relates to what is known in typical mammals including humans and mice.

## Author Contributions

The author confirms being the sole contributor of this work and has approved it for publication.

## Funding

Wellcome Trust Investigator Award for Science 110106/A/15/Z, currently awarded to University of Edinburgh, which supports my labs in Edinburgh and Cambridge.

## Conflict of Interest

The author declares that the research was conducted in the absence of any commercial or financial relationships that could be construed as a potential conflict of interest.
